# Adverse events of nucleos(t)ide analogues for chronic hepatitis B: a systematic review

**DOI:** 10.1007/s00535-020-01680-0

**Published:** 2020-03-17

**Authors:** Raquel Scherer de Fraga, Victor Van Vaisberg, Luiz Cláudio Alfaia Mendes, Flair José Carrilho, Suzane Kioko Ono

**Affiliations:** 1 Department of Gastroenterology, University of São Paulo School of Medicine, Av. Dr. Enéas Carvalho de Aguiar, 255 ICHC, 9th floor, Office 9159, São Paulo, SP 05403-000 Brazil; 2 Division of Clinical Gastroenterology and Hepatology, Hospital das Clínicas, University of São Paulo School of Medicine, São Paulo, SP Brazil; 3 IMED School of Medicine, Passo Fundo, RS Brazil

**Keywords:** Chronic hepatitis B, Nucleotide/nucleoside analogues, Adverse events

## Abstract

Nucleos(t)ide analogues (NAs) are the main drug category used in chronic hepatitis B (CHB) treatment. Despite the fact that NAs have a favourable safety profile, undesired adverse events (AEs) may occur during the treatment of CHB. Given the eminent number of patients currently receiving NAs, even a small risk of any of these toxicities can represent a major medical issue. The main objective of this review was to analyse information available on AEs associated with the use of NAs in published studies. We choose the following MesH terms for this systematic review: chronic hepatitis B, side effects and treatment. All articles published from 1 January 1990 up to 19 February 2018 in MEDLINE of PubMed, EMBASE, the Cochrane Library and LILACS databases were searched. A total of 120 articles were selected for analysis, comprising 6419 patients treated with lamivudine (LAM), 5947 with entecavir (ETV), 3566 with tenofovir disoproxil fumarate (TDF), 3096 with telbivudine (LdT), 1178 with adefovir dipivoxil (ADV) and 876 with tenofovir alafenamide (TAF). The most common AEs in all NAs assessed were abdominal pain/discomfort, nasopharyngitis/upper respiratory tract infections, fatigue, and headache. TAF displays the highest density of AEs per patient treated among NAs (1.14 AE/treated patient). In conclusion, treatment of CHB with NAs is safe, with a low incidence of AEs. Despite the general understanding TAF being safer than TDF, the number of patients treated with TAF still is too small in comparison to other NAs to consolidate an accurate safety profile. PROSPERO Registration No. CRD42018086471

## Introduction

An estimated 257 million people globally are living with chronic hepatitis B (CHB) infection, according to the World Health Organization in 2018 [1]. Treatment’s main goals in CHB are to halt disease progression and prevent disease-related complications, achieved by suppression of hepatitis B virus (HBV) DNA replication [2]. To the present date, CHB treatment is either based on nucleos(t)ide analogue (NA) or on interferon IFNα, currently pegylated (PegIFNα) [3, 4]. NAs that have been approved for HBV treatment in humans include lamivudine (LAM), adefovir dipivoxil (ADV), entecavir (ETV), telbivudine (LdT), tenofovir disoproxil fumarate (TDF) and tenofovir alafenamide (TAF), and can be classified into those associated with low barrier against HBV resistance (LAM, ADV, LdT) and those with high barrier to HBV resistance (ETV, TDF, TAF) [3–5]. The main advantage of treatment with a potent NA with high barrier to resistance (i.e., ETV, TDF, TAF), considered to be the first-line treatment for CHB, is its predictable high long-term antiviral efficacy leading to undetectable HBV DNA levels in the vast majority of compliant patients as well as its good safety profile [3–5]. Moreover, it has been shown that NAs can improve the liver fibrosis and reduce the hepatocarcinogenesis in patients with CHB [6–8].

A significant number of patients has been treated with NAs to date, having increased the experience with their efficacy, resistance and safety profile over the years. Despite the fact that NAs have a favourable safety profile [3, 4], undesired adverse events (AEs) may occur during the treatment of CHB infection. Given the eminent number of patients currently receiving NAs, even a small risk of any of these toxicities can be translated into a major medical issue.

The main objective of this systematic review is to analyse available information in published studies on AEs associated with Nas’ use in adults.

## Methods

### Eligibility criteria

The following research questions were addressed:

- What are the most common AEs with the use of NAs in the CHB treatment?
- Is there any difference in the incidence of AEs between the different NAs?
- Do patients receiving TAF have fewer AEs compared to TDF?

A PICO model was constructed (participants, interventions, control and outcome):

#### Participants

- Adults > 18 years old diagnosed with HBV infection.

#### Interventions

- Antiviral therapy with NAs (LAM, ADV, LdT, ETV, TDF or TAF).

#### Control

- We used only the data for the currently approved dose, i.e. LAM 100 or 150 mg; ADV 10 mg; LdT 600 mg; ETV 0,5 or 1,0 mg; TDF 300 mg; TAF 25 mg.
- Studies based only on drug-combination regimens were excluded due to difficulties in evaluating cause–effect relationship.
- Studies with both single drug arm and drug-combinations arm; only the single drug arm were included in the analysis.

#### Outcome measure

- Adverse events (AEs).

### Exclusion criteria

We excluded studies whose patients presented acute HBV infection, acute liver failure, decompensated cirrhosis, pregnancy, hepatitis C or D or human immunodeficiency virus (HIV) coinfection, schistosomiasis infection; patients receiving corticosteroids, chemotherapy, or immunosuppressive therapy; transplant recipients; and hemodialysis patients. Likewise, studies that did not report AEs or stated "no serious adverse events" or “no significant difference in side effects between groups” with no further AEs description were excluded.

### Literature search strategy

**Figure1:**
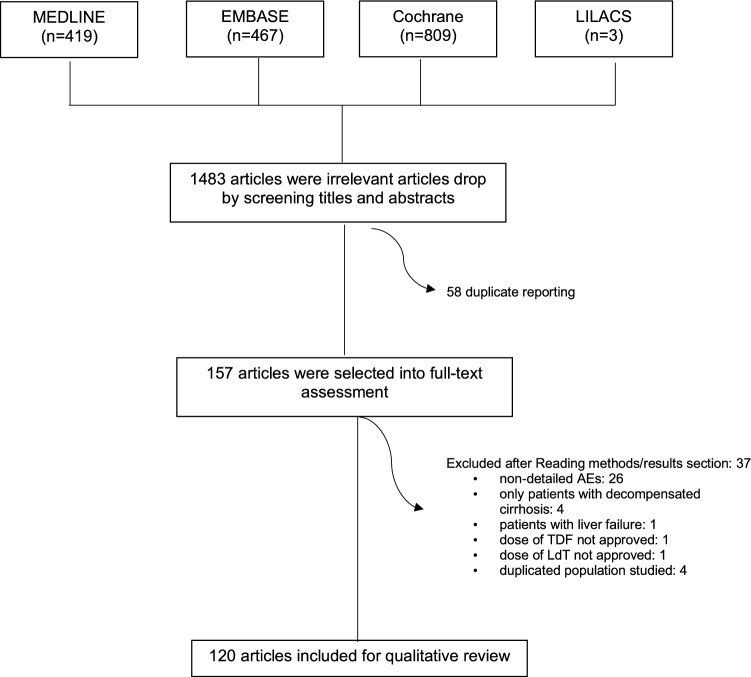
Flowchart of study selection. *MEDLINE* Medical Literature Analysis and Retrieval System Online, *EMBASE* Excerpta Medica Database, *Cochrane* The Cochrane Library, *LILACS* Literatura Latino–Americana e do Caribe em Ciências da Saúde, *AEs* adverse events, *TDF* tenofovir disoproxil fumarate, *LdT* telbivudine

This study was performed according to the PRISMA statement [9]. We chose the following MesH terms: chronic hepatitis B, side effects and treatment. We reviewed all articles published from 1 January 1990 up to 19 February 2018 in MEDLINE of PubMed, EMBASE, the Cochrane Library and LILACS databases and included studies published in English language. Since the NAs have a good safety profile with a small percentage of AEs, we enrolled both observational (i.e. cohort, case–control and cases series) and randomized controlled trials (RCTs) as a search strategy for maximizing AEs sensitivity. All the references identified were managed by Endnote. The flowchart in Fig. 1 shows the process of review of publications. We followed an established protocol which had been registered in PROSPERO (International prospective register of systematic reviews) [10], and the record is available on https://www.crd.york.ac.uk/prospero/ (Registration No. CRD42018086471).

### Data collection and quality assessment

The following data were extracted from included studies: study design, country where the study was conducted, first author, publication year, number of participants, inclusion and exclusion criteria, drug dosing regimens and AEs reported. Two reviewers independently performed data extraction (RSF and VVV) and discrepancies were discussed during a consensus meeting.

To facilitate data analysis, AEs were divided into groups, similar to those found in the VigiAccess™ database, as follows: blood and lymphatic system disorders; cardiac disorders; ear and labyrinth disorders; endocrine disorders; eye disorders; gastrointestinal disorders; general disorders; hepatobiliary disorders; infections and infestations; laboratory abnormalities; metabolism and nutrition disorders; musculoskeletal and connective tissue disorders; neoplasms; nervous system disorders; psychiatric disorders; renal and urinary disorders, reproductive system disorders; respiratory disorders; skin and subcutaneous tissue disorders.

## Results

### Studies

A total of 1698 articles were retrieved. Two authors conducted an initial screening and 1483 studies were excluded after reading titles and abstracts. Following the removal of duplicates, 157 full-text articles were assessed for eligibility. Thirty-seven studies were excluded for the following reasons: non-detailed adverse events (26), only patients with decompensated cirrhosis (4), patients with liver failure (1), dose of TDF not approved (1), dose of LdT not approved (1) and duplicated population studied (4). Finally, 120 articles were selected for analysis. The listing of the included reporters and their characteristics are shown in Table 1.

**Table 1:** Studies reported in this review

Authors, year	Country		Patients (*n*)	Drugs	Study design
Koike, 2018 [39]	Japan		110 56	TDF ETV	Randomized, active controlled, double-blind, double-dummy, parallel arm comparation
An, 2017 [40]	Republic of Korea		47 50	ETV LdT	Randomized open-label
Ashgar, 2017 [41]	Saudi Arabia		23 25	PEGIFN ∝ -2a + TDF TDF	Randomized controlled
Du Jeong, 2017 [42]	Republic of Korea		391	TDF	Retrospective observational
Fung, 2017 [43]	Multiple countries		141 139	TDF FTC/TDF	Prospective, randomized, double-blind, double-dummy
Lee, 2017 [44]	Republic of Korea		56 64	ETV LAM	Phase 4, randomized
Luo, 2017 [45]	China		91 93	LdT ETV	Prospective “real-life”
Rodríguez, 2017 [46]	Spain		22 24	TDF LAM + ADV	Phase 4, prospective, randomized, open, controlled
Yang, 2017 [47]	China		107 115	LAM + vaccine LAM + placebo	Double-blind, randomized, placebo-controlled
Wu, 2017 [48]	Taiwan		106 313	TDF ETV	Retrospective observational
Ahn, 2016 [49]	USA		658	ETV	Observational, retrospective cohort (“real world”)
Buti, 2016 [29]	Multiple countries		285 140	TAF TDF	Randomized, double-blind, phase 3, non-inferiority
Chan, 2016 [30]	Multiple countries		581 292	TAF TDF	Randomized, double-blind, non-inferiority
Huang, 2016 [50]	China		79 45	TDF TDF + NAs	Retrospective cohort
Lim, 2016 [51]	Republic of Korea		45 45	TDF TDF + ETV	Randomized open-label
Marcellin, 2016 [52]	France		440	TDF	Non-interventional, prospective
Marcellin, 2016 [53]	Multiple countries		185 185 186 184	PEGIFN ∝ -2a TDF PEGIFN ∝ -2a + TDF PEGIFN ∝ -2a + TDF16 w + TDF alone 32 w	Randomized open-label, controlled
Shen, 2016 [54]	China		65 65	LdT ETV	Prospective randomized
Zhang, 2016 [55]	China		99 97	ETV LdT	Prospective cohort
Agarwal, 2015 [56]	UK		10 10 11 10 10	TAF 8 mg TAF 25 mg TAF 40 mg TAF 120 mg TDF	Randomized open-label, phase 1b
Alsohaibani, 2015 [57]	Saudi Arabia		68	TDF	Retrospective, observational
Hou, 2015 [58]	China		257 252	TDF ADV	Randomized controlled
Hou, 2015 [59]	China		57	LdT	Cohort
Huang, 2015 [60]	China		33 65	TDF ETV	Retrospective, observational
Jia, 2015 [61]	China		68 68	ADV + LAM ETV	Case–control (prospective)
Kim, 2015 [62]	Republic of Korea		52	TDF	Retrospective observational
Kim, 2015 [63]	Republic of Korea		61 90	LdT ETV	Retrospective observational
Kwon, 2015 [64]	Republic of Korea		39 42	TDF ETV	Retrospective observational
Marcellin, 2015 [65]	Multiple countries		50 54 55	PEGIFN ∝ -2a + LdT PEGIFN ∝ -2a LdT	Randomized,open-label
Yuen, 2015 [66]	Republic of Korea		31 28 30	Besifovir 90 mg Besifovir 150 mg ETV	Randomized open-label, phase 2b
Ahn, 2014 [67]	Republic of Korea		411	TDF	Retrospective, observational
Berg, 2014 [68]	France, Germany, USA		53 52	TDF TDF/FTC	Randomized, double-blind
Chan, 2014 [69]	Multiple countries		62 64	TDF/FTC TDF	Randomized, double-blind, phase 2
Fung, 2014 [70]	Multiple countries		141 130	TDF TDF/FTC	Randomized, double-blind
Jia, 2014 [71]	China		167 165	LdT LAM	Randomized, phase 3
Lai, 2014 [72]	Hong Kong			Besifovir 90 mg Besifovir 150 mg ETV	Randomized open-label, phase 2b
Leung,2014 [73]	China Germany Switzerland		16 14 16	LdT TDF LdT + TDF	Randomized, open-label
Ozaras, 2014 [74]	Turkey		121 130	TDF ETV	Cohort
Pan, 2014 [75]	USA		90	TDF	Open-label, single-arm, phase 4
Sun, 2014 [76]	China		300 299	LdT + ADV LdT	Randomized, open-label, controlled
Du, 2013 [77]	China		25 25	LAM + ADV ETV	Prospective, randomized (pilot study)
Gwak, 2013 [78]	Republic of Korea		50 58	Clevudine ETV	Comparative retrospective
Hou, 2013 [79]	China		2600 (54 patients excluded of analysis – decompensated cirrhosis)	ETV	Prospective, observational cohort
Li, 2013 [80]	China		14 14	LAM (test) LAM (branded reference)	Randomized, open-label
Li,2013 [81]	China		42	LdT	Open-label, single-arm
Lian, 2013 [82]	China		60 60	ADV + LAM ETV	Prospective case–control
Lu, 2013 [83]	China		30 28	LdT + ADV ETV	Randomized open-label
Luo, 2013 [84]	China		230	ETV	Retrospective observational
Marcellin, 2013 [85]	Multiple countries		389 196	TDF ADV followed TDF	Randomized, open-label
Wang, 2013 [86]	China		30 25	LAM + ADV ETV	Randomized open-label
Butti, 2012 [87]	Spain		190	ETV	Retrospective, observational
Heo, 2012 [88]	Republic of Korea		36 36	ETV LAM	Randomized open-label phase 4
Lok, 2012 [89]	Multiple countries		198 186	ETV + TDF ETV	Randomized open-label phase 3b
Gane, 2011 [90]	Multiple countries		389	LdT	Open-label, single-arm
Patterson, 2011 [91]	Australia		38 22	TDF TDF + LAM	Prospective open-label
Perrillo, 2011 [92]	Multiple countries		48 94	LAM + placebo LAM + ADV	Randomized open-label
Safadi, 2011 [93]	Multiple countries		122 124	LdT LAM	Randomized, double-blind, phase 3b
Shin, 2011 [94]	Republic of Korea		109 283	clevudine ETV	Comparative retrospective
Wang, 2011 [95]	China		28 25	LAM LAM + ADV	Prospective controlled
Wang, 2011 [96]	China		31 40	LAM + ADV ETV	Prospective case–control
Berg, 2010 [97]	Multiple countries		52 53	FTC/TDF TDF	Randomized, double-blind, double-dummy
Karino, 2010 [98]	Japan		82	ETV	Open-label, single-arm
Kim, 2010 [99]	Republic of Korea		24 44 36	ETV ADV ADV + LAM	Retrospective cohort
Kim, 2010 [100]	Republic of Korea		55 73	Clevudine ETV	Retrospective cohort
Suh, 2010 [101]	Germany Republic of Korea		23 21	LdT ETV	Open-label, parallel-group, randomized (phase 3b)
Yokosuka, 2010 [8]	Japan		167	ETV	Open-label, single-arm
Zheng, 2010 [102]	China		65 66	LdT ETV	Open-label randomized
Chang, 2009 [103]	Multiple countries		354 355	ETV LAM	Randomized, double-dummy
Kobashi, 2009 [104]	Japan		32 34	ETV 0.1 ETV 0.5	Randomized, double-blind
Liaw, 2009 [105]	Multiple countries		680 687	LdT LAM	Randomized, double-blind, phase 3
Shindo, 2009 [106]	Japan		35 34 34 34	ETV 0.01 mg ETV 0.1 mg ETV 0.5 mg LAM 100 mg	Randomized, double-blind
Yao, 2009 [107]	China		110 329	Placebo/LAM LAM/LAM	Randomized, double-blind
Hou, 2008 [108]	China		167 165	LdT LAM	Randomized, double-blind
Marcellin, 2008 [109]	Multiple countries		426 215	TDF ADV	Randomized, double-blind, phase 3
Marcellin, 2008 [110]	Multiple countries		65	ADV	Open-label, single-arm *
Sung, 2008 [111]	Multiple countries		57 54	LAM LAM + ADV	Randomized, double-blind
Suzuki, 2008 [112]	Japan		41 43	ETV 0.5 ETV 1.0	Randomized, double-blind
Chan, 2007 [113]	Multiple countries		45 44 46	LdT ADV ADV + LdT	Open-label trial
Gish, 2007 [114]	Multiple countries		355 354	LAM ETV	Randomized, double-blind, double-dummy
Lai, 2007 [115]	Multiple countries		680 687	LdT LAM	Randomized, double-blind, phase 3
Lim, 2007 [116]	Multiple countries	Caucasian	142	ADV	Phase 3, randomized, double-blind, placebo controlled
			100	Placebo	
		Asian	138	ADV	Phase 3, randomized, double-blind, placebo controlled
			121	Placebo	
Rapti, 2007 [117]	Greece		14 28	ADV ADV + LAM	randomized controlled study
Ren, 2007 [118]	China		21 21 19	LAM ETV 0.5 mg ETV 1.0 mg	Randomized controlled
Chang, 2006 [119]	Multiple countries		354 355	ETV LAM	Double-blind, double-dummy, randomized, controlled
Hadziyannis, 2006 [120]	Multiple countries		125 62	ADV Placebo	Double-blind phase (96 weeks) + open-label safety and efficacy (144 weeks)
Lai, 2006 [121]	Multiple countries		325 313	ETV LAM	Randomized, double-blind, controlled
Sherman, 2006 [122]	Multiple countries		141 145	ETV LAM	Randomized, double-blind, double-dummy, active controlled
Chan, 2005 [123]	China		50 50	PEGIFN ∝ -2b + LAM LAM	Randomized, controlled, open-label
Chang, 2005 [124]	Multiple countries		42 47 47 45	ETV 1.0 ETV 0.5 ETV 0.1 LAM	Randomized, dose-ranging, phase 2
Lai, 2005 [125]	Multiple countries		19 22 22 21 20	LAM LdT 400 mg LdT 600 mg LAM + LdT 400 mg LAM + LdT 600 mg	Double-blind, randomized, phase 2b
Lau, 2005 [126]	Multiple countries		271 271 272	PEGIFN ∝ -2a PEGIFN ∝ -2a + LAM LAM	Randomized, partially double-blind
Rizzetto, 2005 [127]	Multiple countries		76	LAM	Open-label prospective
Sarin, 2005 [128]	India		38 37	IFN ∝ -2 + LAM LAM	Randomized open-label
Liaw, 2004 [129]	Asia, Australia, United Kingdom		436 215	LAM Placebo	Randomized, double-blind, placebo-controlled, parallel group
Marcellin,2004 [130]	Asia, Europe		177 179 181	PEGIFN ∝ -2a PEGIFN ∝ -2a + LAM LAM	Randomized, partially double-blind
Yao, 2004 [131]	China		322 107	LAM Placebo	Randomized, double-blind, placebo controlled
Ali, 2003 [132]	Iraq		32 30	LAM Placebo	Randomized, placebo controlled
Dienstag, 2003 [133]	Multiple countries		40	LAM	Unblinded, observational
Dienstag, 2003 [134]	Canada, USA, England		63	LAM	Open label, prospective
Marcellin, 2003 [135]	Multiple countries		168 165 161	ADV 10 mg ADV 30 mg Placebo	Randomized, phase 2
Schiff, 2003 [136]	Multiple countries		119 63 53	LAM LAM + IFN ∝ -2b Placebo	Randomized, partially blinded
Lai, 2002 [137]	Multiple countries		54 36 46 41	ETV 0.01 mg ETV 0.1 mg ETV 0.5 mg LAM 100 mg	Randomized, double-blind, dose-ranging
Lai, 2002 [138]	China		50 50	LAM Famciclovir	Randomized, prospective
Mazur, 2002 [139]	Poland		45	LAM	Open-label, prospective
Da Silva, 2001 [140]	Brazil		32	LAM	Open- label, prospective
de Man, 2001 [141]	Multiple countries		8 9 9 8 8	ETV 0.05 mg ETV 0.1 mg ETV 0.5 mg ETV 1.0 mg Placebo	Randomized, placebo-controlled, dose-escalating
Leung, 2001 [142]	China		58	LAM	Open-label, prospective
Montazeri, 2001 [143]	Iran		18 18	LAM LAM + IFN ∝	Randomized, open-label
Hadziyannis, 2000 [144]	Greece		25	LAM	Open-label, single-arm, prospective
Lau, 2000 [145]	USA		27	LAM	Open-label trial, single-arm, prospective
Liaw, 2000 [146]	China		31 101 41 93	LAM 25 mg + placebo LAM 25 mg + LAM 25 mg LAM 100 mg + placebo LAM 100 mg + LAM 100 mg	Randomized, double-blind, placebo controlled
Santantonio, 2000 [147]	Italy		15	LAM	Open-label, single-arm, prospective
Yao, 2000 [148]	China		107 322	Placebo + LAM LAM + LAM	Randomized double-blind placebo controlled
Dienstag, 1999 [149]	USA		66 71	LAM Placebo	Prospective, randomized, double-blind, placebo controlled
Gilson, 1999 [150]	United Kingdom		15 5	ADV Placebo	Randomized, double-blind, placebo controlled, phase I/II
Tassopoulos, 1999 [151]	Multiple countries		60 64	LAM Placebo	Placebo controlled, double-blind, randomized
Lai, 1998 [152]	China		143 142 72	LAM 100 mg LAM 25 Placebo	Randomized, double-blind
Lai, 1997 [153]	China		12 12 12 6	LAM 25 mg LAM 100 mg LAM 300 mg placebo	Randomized, placebo controlled
Nevens, 1997 [154]	Europe		16 16 19	LAM 25 mg LAM 100 mg LAM 300 mg	Randomized, partially double-blind
Dienstag, 1995 [155]	USA		10 11 11	LAM 25 mg LAM 100 mg LAM 300 mg	Double-blind trial

ADV (adefovir dipivoxil); ETV (entecavir); FTC (emtricitabine); IFN (interferon); LAM (lamivudine); LdT (telbivudine); TDF (tenofovir disoproxil fumarate); TAF (tenofovir alafenamide)

^*^ LTSES (long-term safety and efficacy study)

There were 6419 patients treated with LAM, 5947 treated with ETV, 3566 treated with TDF, 3096 treated with LdT, 1178 treated with ADV and 876 treated with TAF.

Table 2 contains the AEs described in the studies, depending on the drugs used.

**Table 2:** Frequency of AEs reported according to the drug

	LAM	ETV	LdT	ADV	TDF	TAF
Studies	49	35	19	10	26	3
Patients	6419	5947	3096	1178	3566	876
AEs	5554	1086	2302	1426	837	998
AEs/patients^a^	0.87	0.18	0.74	1.2	0.23	1.14
Blood and lymphatic systems disorders	20 (0.4%)	3 (0.3%)	22 (1%)	8 (0.6%)	9 (1.1%)	–
Cardiac disorders	7 (0.1%)	6 (0.6%)	1 (0.1%)	–	–	–
Ear and labyrinth disorders	5 (0.1%)	1 (0.1%)	–	1 (0.1%)	–	–
Endocrine disorders	–	–	1 (0.1%)	–	1 (0.1%)	–
Eye disorders	–	1 (0.1%)	6 (0.3%)	1 (0.1%)	–	–
Gastrointestinal disorders	**1116 (20.1%)**	**102 (9.4%)**	**405 (17.6%)**	**244 (17.1%)**	**128 (15.3%)**	**227 (22.7%)**
General disorders	**811 (14.6%)**	**77 (7.1%)**	**214 (9.3%)**	157 (11%)	**82 (9.8%)**	53 (5.3%)
Hepatobiliary disorders	66 (1.2%)	–	–	–	9 (1.1%)	–
Infections and infestations	**871 (15.7%)**	**231 (21.3%)**	**650 (28.2%)**	**260 (18.2%)**	**110 (13.1%)**	**175 (17.5%)**
Laboratory abnormalities	650 (11.7%)	**218 (20.1%)**	**347 (15.1%)**	**179 (12.6%)**	**157 (18.8%)**	**202 (20.2%)**
Metabolism and nutrition disorders	–	–	1 (0.1%)	–	6 (0.7%)	19 (1.9%)
Musculoskeletal and connective tissue disorders	171 (3.1%)	24 (2.2%)	186 (8.1%)	109 (7.6%)	30 (3.6%)	25 (2.5%)
Neoplasms	14 (0.3%)	11 (1%)	7 (0.3%)	–	7 (0.8%)	–
Nervous system disorders	**669 (12%)**	**193 (17.8%)**	**254 (11%)**	**216 (15.1%)**	**66 (7.9%)**	**86 (8.6%)**
Psychiatric disorders	73 (1.3%)	2 (0.2%)	1 (0.1%)	–	4 (0.5%)	–
Renal and urinary disorders	1 (0.02%)	17 (1.6%)	2 (0.1%)	6 (0.4%)	57 (6.8%)	**111 (11.1%)**
Reproductive system disorders	1 (0.02%)	1 (0.1%)	–	–	–	–
Respiratory disorders	**696 (12.5%)**	19 (1.7%)	140 (6.1%)	**200 (14%)**	21 (2.5%)	54 (5.4%)
Skin and subcutaneous tissue disorders	47 (0.7%)	2 (0.2%)	–		15 (1.8%)	–
Serious AEs	271 (4.9%)	131 (12.1%)	53 (2.3%)	39 (2.7%)	82 (9.8%)	37 (3.7%)
Drug discontinuation	62 (1.1%)	33 (3%)	11 (0.5%)	2 (0.1%)	47 (5.6%)	9 (0.9%)
Death	3 (0.1%)	9 (0.8%)	1 (0.1%)	–	4 (0.5%)	–

In each column, the five AEs most often reported were scored in bold. The percentage in parentheses refers to the percentage relative to the total number of AEs reported in each drug

*ADV* adefovir dipivoxil, *AEs* adverse events, *ETV* entecavir, *LAM* lamivudine, *LdT* telbivudine, *TDF* tenofovir disoproxil fumarate, *TAF* tenofovir alafenamide

^a^Mean number of adverse events per treated patient

Neoplasms were documented in 39 patients, with hepatocellular carcinoma being the most frequent– 67% (*n*. 26: LAM-8; ETV-9; TDF-3; LdT-6) [Table 2]. None of the cases were related to NAs use.

### Lamivudine (LAM)

**Fig. 2:**
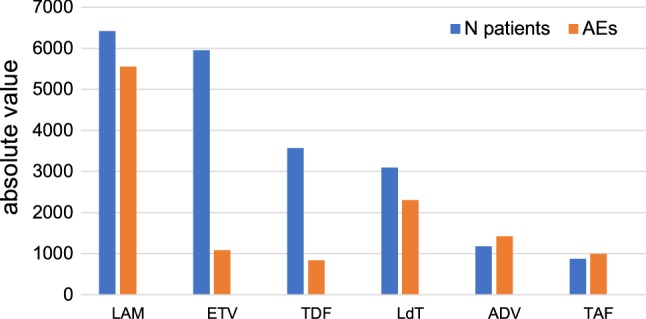
Number of patients treated and absolute value of adverse events reported for each drug. *LAM* lamivudine, *ETV* entecavir, *TDF* tenofovir disoproxil fumarate, *LdT* telbivudine, *ADV* adefovir dipivoxil, *TAF* tenofovir alafenamide

In studies using 100 mg of LAM, a total of 5554 AEs were reported (0.87 AE/patient treated) [Fig. 2, Table 2].

The most frequent AEs reported were gastrointestinal disorders (20.1%), infections and infestations (15.7%), general disorders (14.6%), respiratory disorders (12.5%) and nervous system disorders (12%) [Table 2].

Among gastrointestinal events, the most reported were abdominal pain or discomfort (*n* = 411).

The most commonly described infections were upper respiratory tract infection (*n* = 413).

General disorders included nonspecific symptoms, with asthenia/fatigue being the most reported (*n* = 672).

The most noticed neurological event was headache (*n* = 509). Regarding respiratory problems, viral respiratory infections were the most reported (*n* = 177).

Hepatic enzyme increase was the most documented laboratory abnormality (*n* = 473).

Although rhabdomyolysis was not described, 70 cases of elevated creatine kinase (CK) were documented, but did not lead to drug withdrawal.

### Entecavir (ETV)

In the studies using ETV (0.5 or 1.0 mg), a total of 1086 AEs were reported (0.18 AE/patient treated) [Fig. 2, Table 2].

The most frequent AEs reported were infections and infestations (21.3%), laboratory abnormalities (20.1%), nervous system disorders (17.8%), gastrointestinal disorders (9.4%) and general disorders (7.1%) [Table 2].

Nasopharyngitis was the most frequent infection (*n* = 210). Regarding laboratory abnormalities, ALT elevation was the most reported (*n* = 117).

Headache corresponded to 95% of the nervous system disorders (*n* = 185). Among gastrointestinal disorders, diarrhea was the most common (*n* = 62). Of the general disorders, fatigue was the most reported (*n* = 71). CK elevation has been described in 49 patients.

### Telbivudine (LdT)

In the studies using LdT (600 mg), a total of 2302 AEs were reported (0.74 AE/patient treated) [Fig. 2, Table 2].

The most frequent AEs reported were infections and infestations (28.2%), gastrointestinal disorders (17.6%), laboratory abnormalities (15.1%), nervous system disorders (11%) and general disorders (9.3%) [Table 2].

Nasopharyngitis was the most frequent infection (*n* = 295). Among gastrointestinal disorders, diarrhea was the most common (*n* = 114). Regarding laboratory abnormalities, CK elevation was the most reported (*n* = 211).

Headache corresponded to 72% of the nervous system disorders (*n* = 183). Of the general disorders, fatigue was the most reported (*n* = 71).

### Adefovir dipivoxil (ADV)

A total of 1426 AEs was documented in studies with 10 mg of ADV (1.2 AE/treated patient) [Fig. 2, Table 2].

The most frequent AEs reported were infections and infestations (18.2%), gastrointestinal disorders (17.1%), nervous system disorders (15.1%), respiratory disorders (14%) and laboratory abnormalities (12.6%).

Nasopharyngitis was the most frequently described infection (*n* = 181). Regarding gastrointestinal disorders, the most common was abdominal pain (*n* = 115). Among neurological alterations, headache was the most described (*n* = 185). Regarding respiratory problems, flu syndrome was the most reported (*n* = 96).

ALT elevation was the most frequently described laboratory abnormality (*n* = 90).

CK elevation has been described in 22 patients.

### Tenofovir disoproxil fumarate (TDF)

A total of 837 AEs were documented in studies with 300 mg of TDF (0.23 AE/treated patient) [Fig. 2, Table 2].

The most frequent AEs reported were laboratory abnormalities (18.8%), gastrointestinal disorders (15.3%), infections and infestations (13.1%), general disorders (9.8%) and nervous system disorders (7.9%).

Creatinine elevation was the most frequently described laboratory abnormality (*n* = 30). Regarding gastrointestinal disorders, the most common was nausea (*n* = 44). Nasopharyngitis was the most frequently described infection (*n* = 51). Fatigue was the most reported symptom in the general disorders section (*n* = 44).

Among neurological alterations, headache was the most described (*n* = 54). CK elevation has been described in 13 patients.

When evaluating renal and urinary disorders, 24 cases of urine erythrocytes and 2 cases of urine glucose were reported.

### Tenofovir alafenamide (TAF)

A total of 998 AEs were reported in studies with 25 mg TAF (1.14 AE/treated patient) [Fig. 2, Table 2].

The most frequent AEs reported were gastrointestinal disorders (22.7%), laboratory abnormalities (20.2%), infections and infestations (17.5%), renal and urinary disorders (11.1%) and nervous system disorders (8.6%).

Regarding gastrointestinal disorders, the most common finding was occult blood in stool (*n* = 63). Among the laboratory abnormalities, the most reported were elevated ALT (*n* = 75) and elevated LDL cholesterol (*n* = 35). Nasopharyngitis was the main infections described (*n* = 89). Concerning the renal/urinary changes, urine erythrocytes (*n* = 68) and urine glucose (*n* = 43) were reported.

Headache was the most reported neurological disorder (*n* = 84). Elevation of CK has been described in 23 patients.

### TDF versus TAF

Tables 3 and 4 summarized data on AEs on bone density and renal disorders, respectively, from two studies comparing TDF and TAF.

**Table 3:** Mean percentage decrease in hip and spine bone mineral density with TDF and TAF in studies comparing the two drugs

Study	Follow-up		TAF	TDF	p
Buti, 2016 [29]	48 weeks	hip	− 0.29%	− 2.16%	< 0.0001
		spine	− 0.88%	− 2.51%	0.0004
Chan, 2016 [30]	48 weeks	hip	− 0.1%	− 1.72%	< 0.0001
		spine	− 0.42%	− 2.29%	< 0.0001

**Table 4:** Mean increase in serum creatinine (Cr) from baseline and the median decrease in estimated glomerular filtration rate (eGFR) with TDF and TAF in studies comparing the two drugs

Study	Follow-up		TAF	TDF	*p*
Buti, 2016 [29]	48 weeks	↑Cr (mg/dl)	0.01	0.02	0.32
		↓eGFR (ml/min)	1.8	4.8	0.004
Chan, 2016 [30]	48 weeks	↑Cr (mg/dl)	0.01	0.03	0.02
		↓eGFR (ml/min)	0.6	5.4	< 0.0001

With regard to bone density, TDF caused greater bone loss in both hip and spine compared to TAF [Table 3].

On the other hand, when analysing the renal AEs, there was no clinically significant difference between the two drugs regarding the elevation of serum creatinine, but there was a greater reduction in the glomerular filtration rate in patients who received TDF [Table 4].

### Drug discontinuation due adverse events

**Fig. 3:**
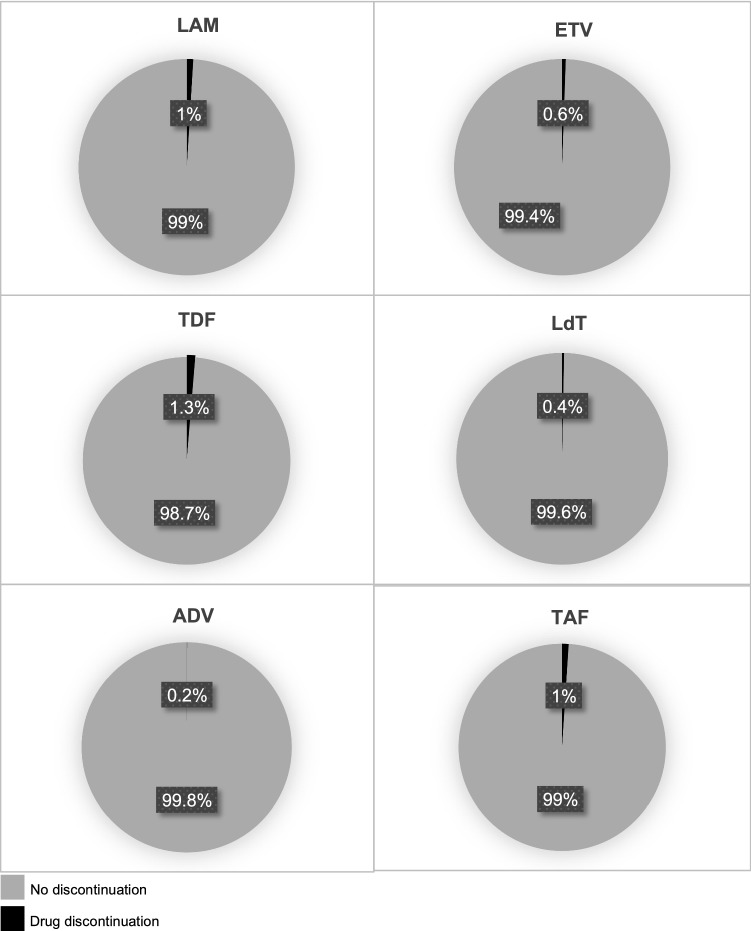
Percentage of drug discontinuation due adverse events for each nucleos(t)ide analogue. *LAM* lamivudine, *ETV* entecavir, *TDF* tenofovir disoproxil fumarate, *LdT* telbivudine, *ADV* adefovir dipivoxil, *TAF* tenofovir alafenamide

In the studies analysed, the percentage of drug discontinuation with LAM, ETV, TDF, LdT, ADV and TAF were, respectively, 1% (*n*. 62), 0.6% (*n*. 33), 1.3% (*n*. 47), 0.4% (*n*. 11), 0.2% (*n*. 2) and 1% (*n*. 9) [Fig. 3].

## Discussion

The aim of CHB treatment was to control viral replication, thereby reducing the risk of complications such as liver failure, cirrhosis and hepatocellular carcinoma. CHB treatment is often based on long-term NAs use, with the following drugs being approved: LAM, ETV, LdT, ADV, TDF and TAF, of which ETV, TDF and TAF are considered to be first-line drugs, due to its potency and high genetic barrier to resistance. Identification of potential associated AEs, even if with low incidence, might be a key factor in improving adherence and outcomes. We performed a systematic literature review of studies that included LAM, ETV, LdT, ADV, TDF and TAF since 1990 and extracted all of the reported AEs from them.

One must be aware upon reading this review, there is no necessarily causation between documented AEs and pharmacological treatment [11]. As hepatitis B infection itself may lead to extrahepatic organ involvement [12], it might be difficult to determine whether extrahepatic manifestations/symptoms are treatment-related or a disease manifestation.

Data collected in this systematic review corroborate with the understanding that serious AEs are rare within the use of NAs. The most common AEs in all NAs assessed were abdominal pain/discomfort, nasopharyngitis/upper respiratory tract infections, fatigue and headache. These symptoms are not uncommon in the general population and, perhaps, these findings are related to their high prevalence among the general population rather than to drug treatment itself.

Extrahepatic AEs may result from mitochondrial toxic effect of NAs [13]. They suppress viral replication by the inhibition of the HBV polymerase enzyme. As NA structures are similar to natural nucleosides, some of these agents can also inhibit human mitochondrial polymerase-γ and cause mitochondrial toxicity [12, 14, 15]. Mitochondrial toxicity was first noticed during HIV treatment with highly active antiretroviral therapy. Because NAs lead to a minimal mitochondrial polymerase-γ inhibition, NAs associated mitochondrial toxicity cases have been rarely reported. All NAs carry a warning of mitochondrial toxicity as part of their prescribing information [12, 14, 15]. Clinical manifestations of mitochondrial toxicity include hematologic disorders, peripheral neuropathy, skeletal and cardiac myopathy, pancreatitis, hepatic failure and lactic acidosis [13, 14, 16]. Among the few AE reported in studies of this systematic review, those that could be correlated to mitochondrial toxicity are CK elevation (70 cases with LAM, 49 with ETV, 211 with LdT, 22 with ADV, 13 with TDF and 23 with TAF)—but without clinical repercussion that required drug suspension; and one case of ETV-related pancreatitis.

Tenofovir disoproxil fumarate (TDF) is a prodrug of tenofovir that was approved as a NA by the United States FDA for use in CHB infection in 2008. TDF is converted to tenofovir by hydrolysis and then phosphorylated by cellular enzymes to tenofovir diphosphate [13]. It is a highly potent inhibitor of HBV DNA replication and recommended as a first-line treatment choice in CHB by the current clinical guidelines due to the absence of drug resistance [17] [18]. Tenofovir has been shown to have a potential nephrotoxic effect in patients with HIV infection who were treated for an especially extended period. However, in clinical trials, nephrotoxicity does not seem to be a major problem in HBV monoinfection [15]. Increases in serum creatinine of > 0.5 mg/dL were reported to be detected in 1% of patient [19]. Another AE concern within TDF use is the bone mass reduction. In randomized clinical trials, a great loss of bone mineral density (BMD) had been well-described in patients with HIV infection treated with TDF [20] [21, 22]. However, tenofovir-related bone fractures were not reported in patients with HBV monoinfection [20]. The exact mechanism of bone toxicity in CHB is not clear. For example, the prevalence of BMD loss in patients receiving tenofovir was similar to those who were not exposed to tenofovir. Tenofovir was reported to be associated with a lower T score only in the hips. Additionally, in this study there was no significant correlation between duration of exposure to tenofovir and reduction in BMD at any side. Additionally, a large retrospective study in Hong Kong demonstrated that BMD reduction remains stable on a plateau from year 4 through year 7 of tenofovir treatment, for both hip and lumbar spine [23]. These data indicate that loss of bone mass is not a progressive event with the use of TDF.

A pro-drug formulation, tenofovir alafenamide (TAF), was recently launched in North America and Europe, being approved for the treatment of CHB in 2016 by the FDA (Food and Drug Administration). The pharmacokinetics of TAF leads to a 6.5-times higher intracellular concentration of the phosphorylated moiety tenofovir diphosphate, and 91% lower serum concentration of tenofovir, compared to TDF [24–26]. Given these pharmacokinetic differences, TAF dose can be far lower: a 25-mg once-daily dose of TAF is bioequivalent to TDF at 300-mg once daily, in terms of tenofovir plasma. Pharmacodynamic studies suggest that the lower tenofovir concentrations in plasma produced by TAF translate to reduced off-target drug exposure, for example, in the kidneys and bones, with implications regarding AEs [27]. TAF is, therefore, predicted to confer the same clinical efficacy as TDF, with potential improvements in its tolerability [27, 28].

In Tables 1 and 2 of this review, we report the results of two studies comparing TDF and TAF (Buti et al. and Chan et al.) [29, 30] concerning renal and bone alteration. The study by Buti et al. had a follow-up of 3 years and Chan et al. had a follow-up of 48 weeks. Both studies suggest that the bone density reduction was greater with the use of TDF, although no drug-related fractures were described. The same occurred with glomerular filtration rate, also with a greater reduction in the groups that received TDF. With these data, we raised two main questions: (1) what is the exact clinical repercussion of these findings? (2) Will such changes remain stable or continue to progress over the years?

Interestingly, renal/urinary changes were the 4th most reported group of AEs among patients on TAF, while the 6th for TDF and the 8th and 12th for ETV and LAM, respectively. Regarding reports for TAF in this group of AEs, there were 43 cases of glycosuria (versus 2 cases with TDF) and 68 cases of urine erythrocytes (versus 24 with TDF). At this time, we do not know the clinical relevance of these findings and whether they may represent any indication of renal tubular damage. Also, the number of patients treated with TAF is markedly lower than the number of patients who received the other NAs. Yet, TAF displays the highest proportion of AEs per patient treated among NAs.

These data fortify the idea that perhaps the greater safety of TAF in relation to TDF may have been overestimated, as already mentioned in the Hill et al. meta-analysis, which compared both drugs in HIV and CHB therapy [31].

It is known that susceptibility to AEs may vary by population. Previously, cases of Fanconi syndrome due to long-term use of adefovir have been reported using adefovir, with a higher incidence in East Asian populations [32]. However, in this review, the incidence of AEs according to ethnicity could not be differentiated. We believe that the low incidence of AEs from NAs makes this differentiation difficult.

Another important point to highlight is that the efficacy of treatments for CHB can be affected by a number of factors, including the development of AEs and poor patient compliance. In fact, a significant number of virological breakthrough may be related to medication nonadherence [33]. Hongthanakorn et al. analysed 148 patients with CHB and demonstrated that 38% of patients who experienced virological breakthrough were not confirmed to have antiviral resistance mutations, suggesting that medication nonadherence may be the cause of the virological breakthrough in these patients [34].

In this review, all drugs had a small percentage of discontinuation due to AEs, which is consistent with the literature. For example, Suzuki et al. reported that 1.3% of patients who were treated with ETV discontinued NA therapy because of AEs. Another study that evaluated LAM, LdT and ETV during the 3-year period found that patients with ETV had the best adherence [35]. This result strengthens the idea of ETV as one of the first-line agents in the treatment of CHB. Nevertheless, it should be emphasized that poor adherence, often still neglected, can have a negative effect on the treatment of chronic hepatitis B, with inadequate viral suppression, increased incidence of cirrhosis and hepatocellular carcinoma, and potential emergence of NAs-resistant [36, 37]. The situation of HBV resistance to NAs in some countries is severe and, to prevent emergence of resistance, NAs with the lowest rate of genotypic resistance should be administered (TDF, TAF or ETV) and adherence reinforced [33, 36–38].

## Conclusion

Treatment of CHB with NAs is safe, with a low incidence of adverse events. The most common AEs with all drugs are abdominal pain/discomfort, nasopharyngitis/upper respiratory tract infections, fatigue and headache. TDF demonstrated a greater reduction in the glomerular filtration rate and bone density of the lumbar spine and hips when compared to TAF. Currently, the number of patients treated with TAF still is too small to consolidate that TAF is really safer than TDF.
